# Taking an Impression Where the Aveolar Ridge Is Soft in Places

**Published:** 1904-06

**Authors:** G. F. Belden

**Affiliations:** Toronto, Can.


### TAKING AN IMPRESSION WHERE THE AVEOLAR RIDGE IS SOFT IN PLACES.

By G. F. Belden, Toronto, Can.

Just one other thing this evening, and that is taking an impression where the aveolar ridge is soft in places. Select cup as before, take full inpression in compound, using the same care in pressing up at the back of the cup; when hard remove, chill and dry; opposite soft part or parts cut wax completely out, then mix some plaster as before directed, placing in enough to fill up portion cut away, and a little spread thinly over the rest of the impression. Insert quickly in the mouth, press firmly to place and opposite soft parts press gently with the finger, also having patient pull down cheeks and lips as before directed—by doing this the impression can be pushed firmly into position without shoving or displacing the soft parts, as the pressure comes on the hard parts of the mouth.

If properly manipulated, you will find when you remove it you have a beautiful impression of the jaw with very little trouble, that would otherwise be a very difficult task in the old way.—*Dominion.*

